# Antibiotic Misuse: An In-Depth Examination of Its Global Consequences and Public Health Challenges

**DOI:** 10.7759/cureus.85941

**Published:** 2025-06-13

**Authors:** Jeevan Nammi, Roshini Pasala, Nikhil Andhe, Ramakanth Vasam, Ausrit Datta Poruri, Ravishankar Raj Sherikar

**Affiliations:** 1 Medicine, Siddhartha Medical College, Vijayawada, IND; 2 General Surgery, Gandhi Medical College, Secunderabad, IND

**Keywords:** antibiotic control, antimicrobial resistance, antimicrobial stewardship program, global epidemiology, public health education

## Abstract

Antibiotic misuse is a growing global threat, driving the rise of antimicrobial resistance (AMR) and endangering our ability to treat common infections and save lives effectively. This paper takes a closer look at how and why antibiotics are being misused in different parts of the world, whether it is through overprescribing by doctors, self-medication by patients, or a lack of strong healthcare regulations. We also explore the serious consequences of AMR, not just for individual patients but for entire health systems and economies. The paper highlights the important roles played by healthcare workers, patients, governments, and the pharmaceutical industry in either contributing to or helping solve this problem. Finally, we discuss practical solutions that have shown promise, such as better education, stricter policies, improved diagnostics, and coordinated action plans. Our goal is to show that reversing antibiotic misuse is not only possible but urgently necessary and that everyone has a role to play in making it happen.

## Introduction and background

**Figure 1:**
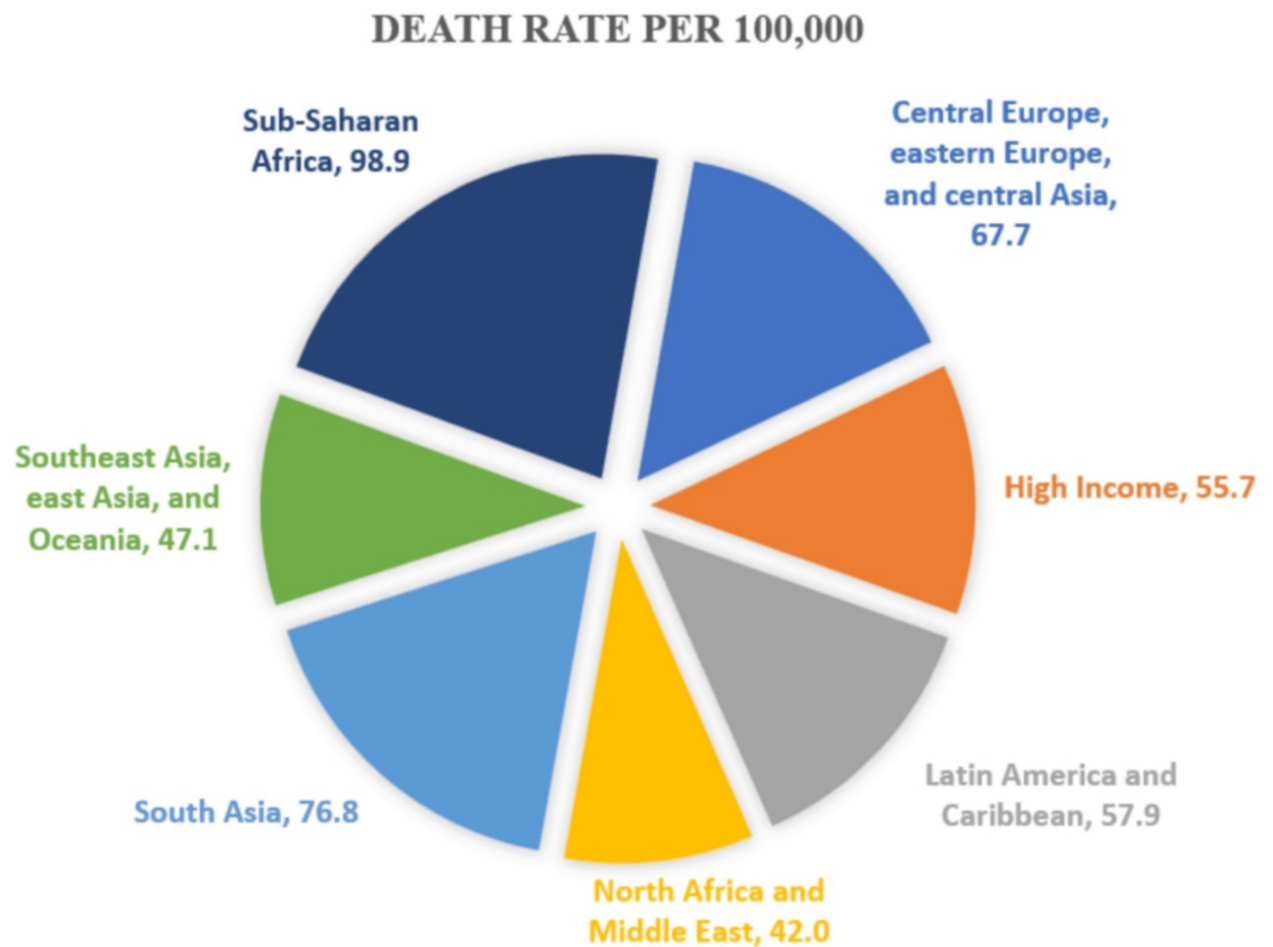
Global death rates attributed to bacterial antimicrobial resistance by GBD super-region, 2019 The High-Income category encompasses high-income regions in North America, Northern and Western Europe, the Asia-Pacific, Latin America, and Australasia. Despite advanced healthcare systems, these areas still experienced a notable mortality burden of 55.7 per 100,000 population Image credit: This is an original image created by the authors Jeevan Nammi and Roshini Pasala using publicly available data [1]

Antibiotics were once miracles of modern medicine; now, they are slipping through our fingers, not from scarcity, but from misuse. Antibiotic misuse refers to the inappropriate or unnecessary use of antibiotics, such as taking them without medical guidance, using them for viral infections, or not completing prescribed courses. This misuse accelerates the development of antimicrobial resistance (AMR), a process where bacteria evolve to resist antibiotic treatment, rendering once-reliable drugs ineffective. AMR has emerged as one of the most pressing global public health and development challenges of the 21st century. In 2019, bacterial AMR was directly responsible for an estimated 1.27 million deaths and contributed to nearly 4.95 million deaths worldwide, underscoring its escalating threat to healthcare systems, disease management, and global health security [1] (the global burden of deaths due to AMR is depicted in Figure 1). While global sepsis mortality has declined in recent decades, AMR-related mortality has continued to rise. Between 1990 and 2019, there was a slight increase in AMR-associated deaths, followed by a modest reduction during the COVID-19 pandemic. AMR could cause 1.91 million attributable deaths and 8.22 million associated deaths annually by 2050. These figures highlight that current efforts fall short of the “10-20-30 by 2030” target, particularly the aim to reduce AMR mortality by 10% [2].

The mechanisms driving resistance are rooted in both clinical and nonclinical settings. Inappropriate prescribing by healthcare professionals, patient self-medication, and the widespread use of antibiotics in agriculture all contribute to the growing crisis. In low- and middle-income countries, these issues are further exacerbated by limited access to diagnostics, the sale of over-the-counter antibiotics, and fragile healthcare systems [3]. This review aims to explore the complex drivers and wide-ranging consequences of antibiotic misuse while also proposing practical, context-sensitive strategies for intervention. By examining the roles of healthcare providers, patients, policymakers, and the pharmaceutical industry, we emphasize the urgent need for coordinated global action to preserve antibiotics' effectiveness for future generations.

## Review

Methods

This narrative review was conducted by the Scale for the Assessment of Narrative Review Articles guidelines to ensure quality and rigor [4]. Relevant literature was sourced from PubMed, Google Scholar, and ScienceDirect using keywords such as “antibiotic misuse”, “antimicrobial resistance”, “self-medication”, and “stewardship”. Peer-reviewed articles, global health reports, and policy documents published between 2010 and 2024 were included based on relevance, credibility, and contribution to the understanding of antibiotic misuse and its global impact.

A thematic analysis approach was used to synthesize evidence across clinical, community, and agricultural settings. The review emphasizes multidisciplinary strategies and the roles of key stakeholders in addressing AMR. No formal meta-analysis was conducted due to the narrative nature of this work.

Discussion

**Figure 2:**
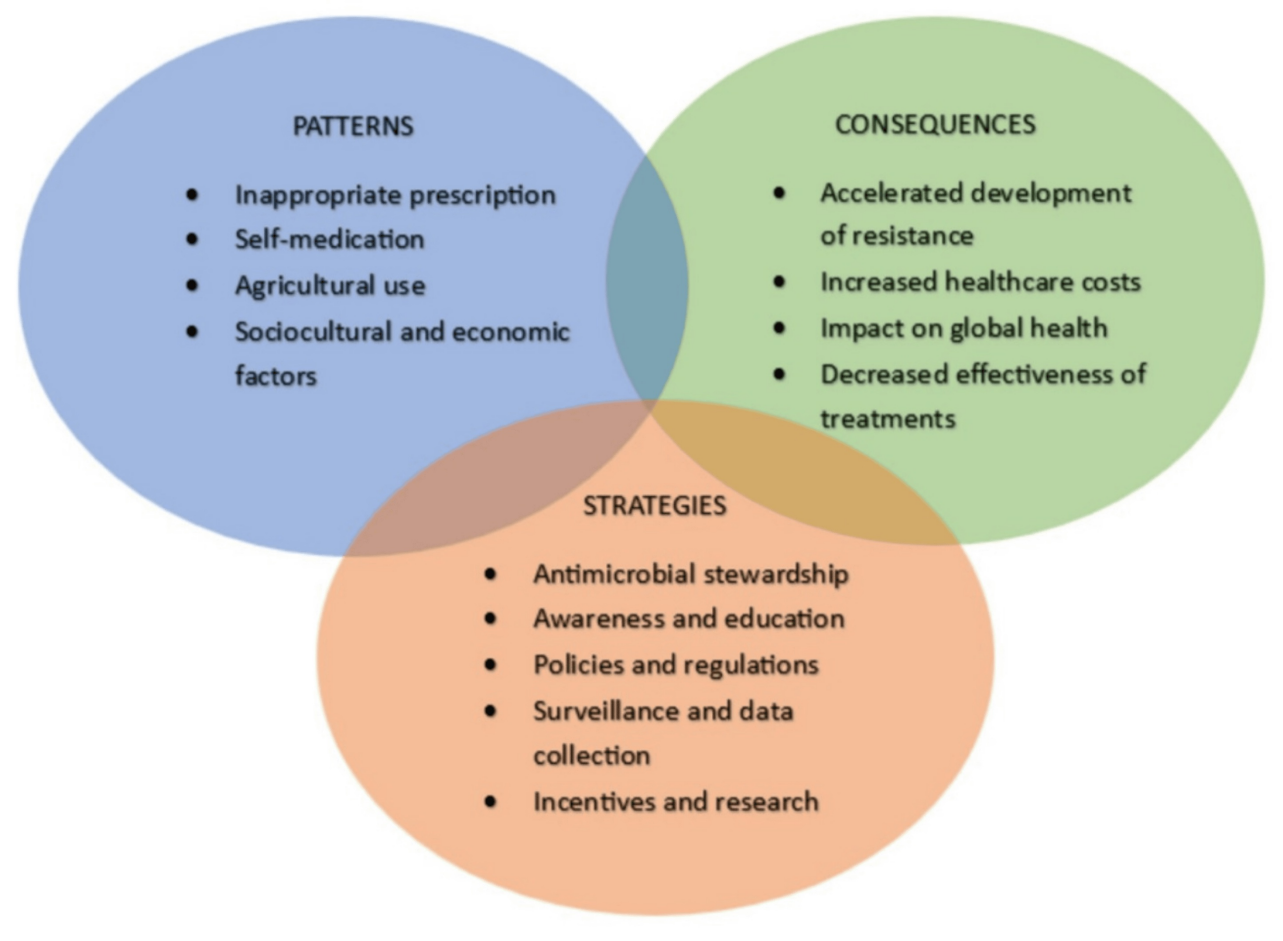
Interconnected landscape of AMR, illustrating the key patterns driving antibiotic misuse, the resulting consequences for global health, and strategic interventions needed to mitigate its impact. The Venn diagram highlights the multidimensional nature of AMR and the need for coordinated, cross-sectoral solutions The Venn diagram illustrates the interplay between the key patterns of antibiotic misuse, its consequences, and strategies to address AMR AMR: antimicrobial resistance Image credit: This is an original diagram created by the authors Jeevan Nammi and Roshini Pasala

Figure 2 visually represents the cross-sectoral nature of AMR, illustrating the interconnected patterns, consequences, and strategies across health systems, communities, and policy frameworks. Each of these components is discussed individually in the subsequent sections of the review to provide a comprehensive understanding of their roles and interrelationships.

Patterns and causes of antibiotic misuse

Inappropriate Prescribing by Healthcare Providers

Inappropriate antibiotic prescribing in clinical settings continues to be a key driver of the global AMR crisis. As the frontline stewards of antibiotic use, healthcare providers often find themselves walking a tightrope between clinical responsibility and everyday challenges, such as limited time, high patient volumes, inadequate diagnostic resources, and pressure to meet patient expectations. These pressures are especially pronounced in developing countries and understaffed healthcare settings, where overburdened clinicians may lack access to up-to-date tools or support systems. Diagnostic uncertainty remains one of the most common dilemmas. When faced with ambiguous symptoms and limited access to reliable diagnostic tools, particularly in underresourced settings, clinicians may choose to “play it safe” by prescribing antibiotics preemptively [5]. This decision, though understandable, can carry long-term consequences. Moreover, patient expectations are a powerful influence; many arrive with the assumption that antibiotics are a cure-all, especially for respiratory or flu-like illnesses. To preserve rapport or avoid conflict, providers may sometimes yield to these expectations, even when aware that antibiotics will offer no benefit [6]. In busy clinics where time is a luxury, rushed consultations can make thoughtful prescribing a challenge. Under pressure to move quickly, clinicians may resort to prescribing antibiotics for nonbacterial or self-limiting conditions, simply to keep the line moving [7]. These everyday compromises, though often made with the best intentions, cumulatively fuel the broader resistance problem we face today.

Self-Medication by Patients

Self-medication with antibiotics remains one of the most persistent and overlooked contributors to antimicrobial misuse, especially in low- and middle-income countries where regulatory oversight is often limited or poorly enforced. In many parts of the world, a person can walk into a pharmacy and purchase antibiotics without consulting a doctor, without any questions asked. This over-the-counter access, particularly common in rural or underserved communities, allows individuals to treat themselves for illnesses like the common cold or flu, which are often viral and do not require antibiotics in the first place [8]. With no medical guidance on the right dosage, how long to take the medication, or whether it is needed at all, people often take antibiotics irregularly, stopping as soon as they feel better, using leftover pills from previous illnesses, or sharing them with family members. These habits, though sometimes born out of necessity or good intentions, create the perfect breeding ground for resistant bacteria to thrive and spread [9]. What is more, self-medication has become normalized in many communities, driven by practical realities: the high cost of healthcare, long travel distances to clinics, and limited understanding of how antibiotics work. Tackling this deeply rooted issue calls for more than just stricter pharmacy laws; it demands culturally sensitive public education campaigns that build trust, dispel myths, and empower people to seek care responsibly. Additionally, in some regions, informal healthcare providers, often the first point of contact in medically underserved communities, may recommend or dispense antibiotics without appropriate knowledge or licensing, further reinforcing misuse patterns. Cultural beliefs and personal experiences also play a role; many people associate antibiotics with fast recovery and may insist on them based on prior perceived effectiveness, regardless of current clinical need. This unchecked misuse not only affects individual outcomes but also poses a broader threat to global health, as resistant pathogens can easily cross borders and undermine medical advances worldwide.

Use in Agriculture

The use of antibiotics in agriculture is a significant yet often overlooked contributor to the growing global AMR crisis. In many livestock farms, antibiotics are commonly administered not just for treating infections but also for promoting growth and preventing diseases in otherwise healthy animals. This routine, nontherapeutic use is a major breeding ground for the development of resistant bacteria, which can then spread to humans in several ways, whether through direct contact with animals, environmental contamination, or consumption of animal products like meat, milk, and eggs [10]. Research has shown that resistant bacteria originating in agricultural settings can find their way into the human microbiome, creating a direct threat to the effectiveness of antibiotics and complicating infection management for human patients [3]. The agricultural sector’s significant role in the rise of AMR calls for a One Health approach, which underscores the interconnectedness of human, animal, and environmental health. In this context, restricting unnecessary antibiotic use in food production, encouraging better farming practices, and increasing veterinary oversight are crucial steps toward protecting both human and animal health. Moreover, improving public awareness of the issue in both the farming community and among consumers can drive more responsible antibiotic use and support the global fight against AMR. For instance, countries with weaker regulatory frameworks in agriculture may see much higher levels of antibiotic use in farming, exacerbating AMR transmission globally.

Sociocultural and Economic Factors

Sociocultural and economic factors significantly drive antibiotic misuse, especially in low- and middle-income countries. Unregulated antibiotic sales and easy access without prescriptions are common due to weak healthcare systems, inadequate regulation, and fragmented pharmaceutical markets [11]. Poverty and limited access to healthcare push individuals toward self-medication and informal vendors as cheaper, quicker options.

Cultural beliefs and misinformation further worsen misuse, with antibiotics often mistakenly used for viral infections like colds or flu. This contributes to growing AMR.

Addressing these issues requires a culturally sensitive, multifaceted approach: enforcing stricter regulation of antibiotic sales, improving access to healthcare in underserved areas, and implementing targeted public education campaigns to correct misconceptions and promote responsible use. Such integrated efforts are crucial to effectively reducing antibiotic misuse and combating resistance in the long term.

Consequences of antibiotic misuse

The misuse of antibiotics has far-reaching consequences for public health, the economy, and medical practice. We delve into the multifaceted effects of antibiotic misuse in the following.

Accelerated Development of AMR

The most immediate and alarming consequence of antibiotic misuse is the rapid acceleration of AMR. When antibiotics are used unnecessarily or inappropriately, whether in humans, animals, or agriculture, they create selective pressure that allows resistant bacteria to survive and multiply. These resistant strains can spread quickly, rendering once-effective treatments obsolete and making infections increasingly difficult to treat. This phenomenon is particularly concerning for vulnerable populations, including immunocompromised individuals, the elderly, and patients undergoing complex medical procedures, all of whom are at heightened risk of treatment failure. As resistance continues to rise, so too does the likelihood of prolonged illness, increased transmission rates, and, ultimately, higher mortality [7,12]. The impacts of AMR extend far beyond individuals, placing a significant burden on healthcare systems, with prolonged hospital stays and more intensive care required to treat resistant infections.

Moreover, the diminishing effectiveness of antibiotics compromises the safety and success of critical medical interventions, including surgeries, cancer treatments, and organ transplants. Procedures that were once routine and relatively safe are becoming increasingly risky in the face of rising antibiotic resistance. For instance, patients undergoing chemotherapy are particularly vulnerable to infections, and with fewer effective antibiotics available, even common postsurgical infections may lead to severe complications and death. The growing shortage of effective treatments also increases healthcare costs, as more expensive and often less effective alternatives must be used.

Suppose immediate action is not taken to curb antibiotic misuse. In that case, AMR threatens not only to reverse decades of medical progress but also to become one of the leading causes of death worldwide, pushing us back to a time when minor infections were often fatal. This makes it all the more crucial for global and local communities to come together and address the root causes of AMR, prioritizing prevention, regulation, and responsible antibiotic use to safeguard future generations.

Increased Healthcare Costs

The financial burden of AMR is substantial and continues to grow, posing a significant challenge to both healthcare systems and economies worldwide. As infections become more resistant to treatment, they demand longer durations of care, more expensive or toxic alternative medications, and extended hospital stays. This not only leads to a sharp increase in direct medical costs, including diagnostics, drugs, and intensive care, but also contributes to indirect costs, such as lost productivity due to prolonged illness or death [12]. In resource-limited settings, the added financial strain often forces families to pay out-of-pocket for treatments, further exacerbating economic hardship and perpetuating a cycle of poverty and poor health outcomes [13].

On a global scale, the financial implications of AMR are even more concerning. The World Bank has issued warnings that if AMR is left unaddressed, it could result in a reduction of 1%-3.8% in annual global gross domestic product by 2050. These projections highlight the far-reaching economic consequences of AMR, underscoring that AMR is not just a medical issue but a profound economic threat. Beyond the healthcare sector, the economic ripple effects could impact trade, labor markets, and productivity, especially in regions already facing economic challenges. These stark projections make it clear that combating AMR requires urgent and sustained investment in prevention, surveillance, and policy reform. By addressing the root causes of AMR, we can mitigate its financial impact, protect public health, and safeguard global economic stability.

Impact on Global Health Security

AMR is a growing threat to global health security, particularly in the context of pandemics and emerging infectious diseases. The overuse of antibiotics, especially during viral infections such as COVID-19, has significantly contributed to the spread of resistant bacteria, complicating efforts to control infectious diseases during health crises. The widespread misuse of antibiotics in these situations has led to a dangerous cycle of resistance, making it harder to treat both common and complex infections. Amid pandemics, the presence of resistant pathogens can not only make clinical management more challenging but also prolong recovery times, heightening the risk of secondary infections that further strain overwhelmed healthcare systems.

As AMR continues to spread, it poses a unique challenge for global health, especially as international travel and trade facilitate the rapid transmission of resistant infections across borders. Both high-income and low-income countries are at risk, though resource-limited settings often bear a disproportionate burden due to inadequate healthcare infrastructure and access to treatment. The COVID-19 pandemic has underscored the devastating potential of AMR to derail pandemic response efforts, with resistance undermining the effectiveness of antibiotics used to treat secondary bacterial infections in critically ill patients. This crisis emphasizes the urgent need for a coordinated, global response to control AMR and protect public health, with robust international collaboration on surveillance, policy enforcement, and education to mitigate its spread [14].

Diminished Effectiveness of Medical Procedures

The growing prevalence of AMR threatens the safety and success of essential medical procedures that depend on effective antibiotics, such as surgeries, organ transplants, chemotherapy, and the care of immunocompromised patients. As resistance reduces the effectiveness of these drugs, even routine interventions carry a heightened risk of postoperative infections and complications. This not only endangers patient outcomes but also raises concerns about the future viability of complex medical care. Without reliable antibiotics, once-manageable infections could again become life-threatening, potentially reversing decades of medical progress. The dwindling arsenal of effective treatments leaves healthcare providers with fewer options to protect their most vulnerable patients, underscoring the urgent need to combat AMR [15].

Strategies to mitigate antibiotic misuse

The fight against antibiotic misuse requires a multipronged approach involving various stakeholders, including governments, healthcare providers, pharmaceutical industries, and the public.

Antimicrobial Stewardship Programs

Antimicrobial stewardship programs (ASPs) are crucial for promoting the responsible use of antibiotics and mitigating the rise of AMR. These programs are designed to optimize the selection, dosage, route, and duration of antibiotic therapy to achieve the best clinical outcomes while minimizing potential toxicity and the development of resistance. Effective stewardship involves several key strategies, including evidence-based prescribing, timely de-escalation from broad-spectrum antibiotics to more targeted therapies, and the use of narrow-spectrum antibiotics when appropriate. In addition to these clinical practices, clinician education, regular audits with feedback, microbiological surveillance, and robust institutional policies are essential to maintaining and strengthening stewardship efforts.

By embedding these strategies into everyday clinical practice, ASPs not only help preserve the efficacy of existing antibiotics but also enhance patient safety and reduce unnecessary healthcare costs. Their implementation across diverse healthcare settings, such as hospitals, outpatient clinics, and long-term care facilities, is vital in slowing the spread of multidrug-resistant organisms and ensuring that modern medicine continues to deliver life-saving interventions. ASPs, when properly executed, serve as a cornerstone in the global fight against AMR, preserving the ability to treat infections and preventing the escalation of resistance [11,16].

Public Awareness and Education

Raising public awareness and improving education about AMR are critical steps in reducing antibiotic misuse and cultivating a culture of responsible antimicrobial practices. Misconceptions about antibiotics, such as the widespread belief that they are effective against viral infections, are significant drivers of inappropriate antibiotic use. To address this, comprehensive educational campaigns targeting both the general public and healthcare providers are essential. These efforts could include mass media outreach, school-based programs, community engagement activities, and pharmacist-led counseling at the point of sale.

Incorporating AMR education into routine healthcare interactions ensures that messages about proper antibiotic use are reinforced consistently. Culturally sensitive communication, supported by local influencers, community leaders, and civil society organizations, can significantly enhance message acceptance and ensure that educational efforts resonate with diverse populations. On a broader level, collaboration among healthcare institutions, educational systems, and government bodies is crucial to creating a unified approach to AMR awareness. Standardizing messaging across all levels of society and embedding it into public health strategies will help ensure that the fight against antibiotic resistance is cohesive and sustainable.

Ultimately, strengthening public understanding of AMR is not just about disseminating information; it is about transforming mindsets and empowering individuals to take an active role in combating resistance. When people understand the consequences of misuse, they are more likely to make informed choices and demand better practices from both healthcare providers and policymakers, making AMR education a key tool in global health [17].

Policy and Regulatory Measures

Effective policy and regulatory frameworks are essential in tackling the multifaceted and escalating challenge of AMR. At the heart of this effort lies the need for strong regulations to govern the sale and distribution of antibiotics, ensuring they are dispensed only with valid prescriptions and used responsibly across both human and animal health sectors. This is especially critical in countries where antibiotics are still readily available over the counter, fueling widespread misuse.

National action plans should go beyond broad goals and actively enforce key interventions: mandating ASPs in healthcare institutions, tightening controls on agricultural and veterinary antibiotic use, and establishing standardized protocols for antibiotic prescribing. Equally vital is the development of comprehensive surveillance systems that monitor resistance trends and antibiotic consumption, providing the data needed to fine-tune interventions and respond to emerging threats in real time [18,19].

However, effective regulation cannot follow a one-size-fits-all approach. Policies must be adaptable and sensitive to regional differences in healthcare access, economic capacity, and public awareness. In lower resource settings, for example, regulatory strategies should be paired with investments in healthcare infrastructure and community education to avoid creating barriers to essential treatment.

Collaboration is the cornerstone of sustainable progress. Governments, healthcare professionals, researchers, agricultural stakeholders, and civil society must work in tandem to create a regulatory environment that is not only strict but also inclusive and fair. When supported by education and community engagement, regulatory efforts become more than just rules; they become a shared social contract to preserve antibiotic efficacy for future generations.

Surveillance and Data Collection

Comprehensive surveillance and data collection form the backbone of the global response to AMR. Without reliable, real-time data, efforts to contain resistance risk are reactive rather than proactive. Surveillance systems help identify emerging resistance trends, evaluate the effectiveness of antibiotic stewardship programs, and guide the development of evidence-based treatment protocols. Platforms such as the World Health Organization’s Global Antimicrobial Resistance and Use Surveillance System [20,21] have been instrumental in promoting standardized, cross-border data collection and fostering international collaboration.

To strengthen national and regional surveillance efforts, countries must invest in laboratory capacity, train a skilled workforce, and establish standardized protocols for data gathering and reporting. This includes integrating microbiological, clinical, and epidemiological information to provide a holistic view of how resistance is spreading and its underlying causes. In many low- and middle-income countries, support for infrastructure development and workforce training is crucial to closing gaps in surveillance and ensuring that the data collected is both accurate and actionable.

Transparent, harmonized data sharing is equally important. Global health security depends on the ability of nations to communicate and coordinate in the face of emerging resistance threats. Whether tracking a local outbreak of multidrug-resistant bacteria or responding to global trends in antibiotic misuse, access to reliable data enables swift, informed decision-making.

Ultimately, resistance knows no borders, and neither should our surveillance efforts. Establishing robust data systems not only empowers healthcare providers and policymakers but also bridges inequalities between countries, creating the foundation for a truly united and effective global response to AMR.

Incentivizing Research and Development

Revitalizing the pipeline of new antibiotics is critical to staying ahead of AMR, yet innovation in this field has stalled due to scientific, regulatory, and economic challenges. Antibiotics often yield lower financial returns compared to drugs for chronic conditions, as they are typically used for short durations and stewardship efforts limit their widespread use. To counter this, innovative incentive models have emerged, including "push" mechanisms like grants for early-stage research, and "pull" mechanisms such as market entry rewards and subscription-based models that decouple revenue from sales volume. Global initiatives like Combating Antibiotic-Resistant Bacteria Biopharmaceutical Accelerator and the AMR Action Fund support early development of promising compounds and help advance them through clinical trials. Additionally, fostering public-private partnerships and ensuring equitable access to new antibiotics, especially in low- and middle-income countries, are essential components of a sustainable solution. Research and Development efforts must be underpinned by long-term policy commitments and global collaboration to secure a robust antibiotic arsenal for the future [22,23].

## Conclusions

AMR remains a major global health threat, largely driven by the misuse of antibiotics across clinical, community, and agricultural settings. While the issue has been widely examined, this review offers a more integrated and grounded perspective by highlighting the often-overlooked real-world pressures, such as diagnostic uncertainty, underresourced healthcare systems, cultural beliefs, and regulatory gaps, that influence antibiotic use, particularly in low- and middle-income countries. What distinguishes this review is its focus on translating global knowledge into context-sensitive, cross-sector solutions that are practical and scalable. Rather than reiterating known facts, it calls attention to the interplay between policy, public behavior, and health infrastructure, and advocates for coordinated action through stewardship programs, public education, stronger surveillance, and responsible regulation. Ultimately, addressing AMR demands more than awareness, it requires shared responsibility, inclusive strategies, and urgent, sustained commitment to preserve the effectiveness of antibiotics for future generations.
